# MiR-223-3p regulates erythropoiesis by targeting TGFBR3/Smad signaling pathway in hemoglobin H-Constant Spring disease

**DOI:** 10.1080/07853890.2025.2530690

**Published:** 2025-07-11

**Authors:** Lan Lan, Xinyu Wang, Simeng Zhang, Dan Zeng, Limin Mo, Qiao Feng, Jun Li, Chunjiang Zhu

**Affiliations:** Genetics and Precision Medicine Laboratory, Affiliated Hospital of Guilin Medical University, Guilin, China

**Keywords:** Thalassemia, HbH-CS disease, miR-223-3p, TGFBR3

## Abstract

**Background:**

MicroRNAs (miRNAs) have emerged as regulators of pathogenesis in hematopoiesis by targeting post-transcription mRNA regulation. However, the involvement of miRNAs in hemoglobin H-Constant Spring (HbH-CS) disease remains unclear. The present study analyzed miRNAs differential expression profile between patients with HbH-CS disease and healthy subjects by Arraystar Human small RNA Microarray.

**Methods:**

The differential expression profiles of miRNAs between HbH-CS patients and healthy individuals were analyzed using Arraystar human small RNA microarrays. Quantitative real-time PCR and Western blot were used to detect the mRNA and protein expression. Bioinformatics methods were used to predict the target genes of miRNAs, and the regulatory relationship between miRNAs and target genes was verified by dual-luciferase reporter assay. Cell apoptosis was detected by flow cytometry, and cell proliferative capacity was detected by CC K-8 assay.

**Results:**

MiR-223-3p was significantly down-regulated in HbH-CS patients. Dual-luciferase reporter assay demonstrated that miR-223-3p specifically targeted TGFBR3. The *in vitro* experiments showed that miR-223-3p inversely regulated the expression levels of TGFBR3, Smad2/3, and P-Smad2. We found that overexpression of miR-223-3p in K562 cells significantly promoted cellular proliferation and inhibited cellular apoptosis by suppressing expression of TGFBR3, Smad2/3, and P-Smad2. Moreover, up-regulation of miR-223-3p in induced K562 cells increased the expression levels of hemoglobin and mean corpuscular hemoglobin.

**Conclusion:**

In conclusion, this study indicated that the interaction between miR-223-3p and TGFBR3/Smad signaling pathway might affect erythropoiesis in HbH-CS disease, which can provide more information for the treatment of patients.

## Introduction

Thalassemia is a common genetic hematological disease, which is mainly prevalent in Southern China, South Asia, Northern Africa, the Middle East, and the Mediterranean region. According to the previous literature, about 5% and 1.5% of the population worldwide carry the α- and β-thalassemia allele, respectively [1,2]. Indeed, a higher prevalence of α- and β-thalassemia is reported in Southern China, especially in Guangdong et al. [3].

There are two α-peptide chains and two β-peptide chains in normal hemoglobin of human beings. The mutations in α- or β-globin gene lead to decreased or absence synthesis of α- or β-globin chains, which cause abnormalities in the morphology and function of erythrocytes, resulting in anemia [4,5]. When three of four α-globin genes are defective, the synthesis of α-globin chains is markedly decreased, leading to excess of free β-chain chains with the formation of unstable β-globin tetramer (β4), designated as hemoglobin H (HbH) [6]. HbH disease is characterized by remarkably genetic heterogeneity and generally presents a mild to moderate chronic hemolytic anemia. Hemoglobin H-Constant Spring (HbH-CS) is a common form of nondeletional HbH disease. HbH-CS disease results from compound heterozygosity with both linked α-globin genes deletions and Constant Spring (CS) mutation [7,8]. Some of the patients with HbH-CS disease have severe phenotype and require regular transfusion.

MicroRNAs (miRNAs) are non-coding small molecule RNAs. In recent years, with the development of molecular biology and genomics, accumulating evidence has demonstrated that miRNAs play important roles in hematological diseases [9]. MiRNAs have been found to influence the onset and development of thalassemia by regulating the expression of genes related to hemoglobin synthesis and cellular biological processes. Abnormal expression of miRNA can affect erythropoiesis [9,10]. However, the role of miRNAs in HbH-CS disease is unclear, thus the relationship between miRNAs and HbH-CS disease deserves further investigation. This study aimed to investigate the expression profiles of miRNAs in HbH-CS disease and the roles of these miRNAs on the biological process of erythropoiesis and the synthesis of hemoglobin in HbH-CS disease. It is hoped that this study can provide promising targets and strategies for the treatment of HbH-CS disease, which will be effective for precision medicine and genetic disease treatment.

## Materials and methods

### Study participants

A total of 4 HbH-CS patients (genotype: –^SEA^/α^CS^α) and 4 normal (Hb ≥ 110 g/L) healthy subjects were included in this study. The hematological characteristics of participants are shown in Table 1. The exclusion criteria of the present study were as follows: 1) concomitant with other thalassemia mutations; 2) serum ferritin (SF) ≤ 12ug/ul; 3) received splenectomy; 4) history of blood transfusion within two months; 5) concomitant with infections, neoplasms, and autoimmune disorders. This study was complied with the Declaration of Helsinki Principles and approved by the Ethics Committee of Affiliated Hospital of Guilin Medical University (NO.2020GZRLL-46). The participants were recruited from the Affiliated Hospital of Guilin Medical University and provided written informed consent prior to the start of the study.

**Table 1.** Sample information.

Sample	Sex	HGB	HCT	MCV	MCH	MCHC	RBC
		(g/L)	(%)	(fl)	(pg)	V(g/L)	(*10^12^/L)
(--^SEA^/α^CS^α)							
1	F	94	33.9	72.3	20	277	4.69
2	M	66	27.3	83.2	20.1	242	3.28
3	M	88	31.2	70.9	20	282	4.4
4	M	44	18.7	82	19.3	235	2.28
5	F	95	29.3	75	24.1	323	3.93
6	F	81	29.1	77	21.5	280	3.8
7	M	76	28.7	73	19.4	266	3.92
8	M	92	31.2	66	19.3	294	4.76
9	F	87	32.3	76.5	20.6	269	4.22
10	M	38	10.8	70.7	25.2	357	1.52
11	M	66	23.6	64.9	18.1	280	3.68
12	F	92	31.5	71.1	20.8	292	4.43
13	F	66	26.2	78	19.7	252	3.35
14	F	56	18.2	74.2	22.7	306	2.45
15	M	97	31.6	68.6	21.1	307	4.61
16	M	30	10.7	73.8	20.7	280	1.45
17	F	90	31.6	69.5	19.8	284	4.55
Normal							
1	M	130	39.0	86.9	29	333	4.49
2	M	114	34.5	83.7	27.7	330	4.12
3	M	132	38.7	86.9	30	335	4.85
4	M	122	37.6	88.5	28.7	324	4.25
5	M	165	48.3	83.6	28.5	342	5.75
6	F	126	37	94.7	32.3	341	3.91
7	F	138	41.4	90.2	30	333	4.58
8	M	153	46.4	83.2	27.4	330	5.59
9	F	134	38.4	89.7	31.3	349	4.28
10	F	148	43.2	85.5	29.3	343	5.05
11	F	143	43	86.8	29.5	333	4.84
12	F	136	39.2	84.7	29.4	347	4.63
13	F	136	41.1	92.8	30.7	331	4.43
14	M	141	41.9	87.1	29.3	337	4.81
15	M	138	42.5	81.8	29.8	325	4.62
16	F	130	39.5	82.2	27	329	4.8
17	M	158	46.7	85.1	28.8	338	5.49

HGB, hemoglobin; HCT, red blood cell specific volume; MCV, mean corpuscular volume; MCH, mean corpuscular hemoglobin, MCHC, mean corpuscular hemoglobin concentration; RBC, red blood cell; F, female; M, male.

### RNA extraction from reticulocytes

Peripheral blood samples (10 ml) were collected, and mononuclear cells were isolated by using density gradient centrifugation mononuclear cell separation media (Solarbio, Beijing, China). Then, use the CD71^+^ selection method (CD71 Antibody, anti-human, REAfinity^™^) in combination with the MACS^™^ separation system (Miltenyi Biotech, Auburn, CA, USA) to isolate reticulocytes and nucleated red blood cells from the mononuclear cells. The purity of isolated cells was determined by flow cytometry. RNA was extracted using Nucleozol (macherey-nagel) according to the instructions. RNA integrity and gDNA contamination were determined by denaturing agarose gel electrophoresis. RNA concentration and quality were determined by NanoDrop ND-1000.

### MiRNA microarray analysis

RNA samples were quantified using NanoDrop ND-1000, and integrity assessed with Bioanalyzer 2100 or gel electrophoresis. 100 ng of RNA was dephosphorylated, denatured with DMSO, and labeled with Cy3. The labeled RNA was hybridized on the Arraystar Human small RNA Microarray and scanned with the Agilent Scanner G2505C. Data were processed with Agilent Feature Extraction and GeneSpring GX v12.1. Only probes with Present (P) or Marginal (M) QC flags in at least 4 of 8 samples were kept, with multiple probes for the same RNA aggregated. Differential expression was analyzed using fold change and P-value thresholds. Expression patterns were visualized with heatmaps, scatter plots, and volcano plots in R. Experimental procedures followed those described in the works of Hou, Z., Mahajan, A., Liu, R., and others [11–13].

### MRNA microarray analysis

The experimental procedure followed the methods of Hou et al. [11–13]. The concentration and specific activity of labeled cRNAs were quantified using a NanoDrop ND-1000 spectrophotometer. Each 1 μg of labeled cRNA was fragmented by adding 5 μl of 10 × Blocking Agent and 1 μl of 25 × Fragmentation Buffer, followed by incubation at 60 °C for 30 min. Afterward, 25 μl of 2 × GE Hybridization buffer was added to dilute the cRNA. The hybridization solution (50 μl) was applied to the gasket slide and aligned with the microarray slide. The slides were incubated in an Agilent Hybridization Oven at 65 °C for 17 h. Post-hybridization, the arrays were washed, fixed, and scanned with the Agilent DNA Microarray Scanner. The obtained images were analyzed using Agilent Feature Extraction software, and quantile normalization was performed with GeneSpring GX v12.1. Differentially expressed mRNAs with statistical significance were identified using P-value/FDR filtering, and Fold Change was used for further assessment. Pathway and Gene Ontology (GO) analyses explored the biological roles of differentially expressed mRNAs, while hierarchical clustering and combined analyses were performed using custom scripts.

### Analysis of miRNA expression and hematology-related genes

MiRNAs associated with hematopoietic cell lineage, apoptosis, and cell cycle were searched in the database, which were stratified by Arraystar miRNA expression profiling microarray data filtered according to *p* < 0.05 and log FC >1.5.

### Verification of the differentially expressed miRNA and its related mRNA

The differentially expressed miRNAs were verified by using quantitative real-time PCR (qRT-PCR). cDNA was synthesized from total RNA by using the PrimeScript RT Enzyme Mix I kit (Takara Bio, Shiga, Japan) and the RT kit for fluorescence quantification of miRNA (Takara Bio, Shiga, Japan), respectively. Next, qRT-PCR analysis was performed using TB Green Premix Ex Taq II kit (Takara Bio, Shiga, Japan), according to the manufacturer’s instructions. The levels of the target gene and miRNA expression were normalized to the levels of β-actin and U6 expression, respectively. The sequences of the primers used are showed in Table 2. Relative gene expression was calculated using the comparative 2^−△△Ct^ methods.

**Table 2.** Primer sequences.

Name of primer	Sequences
miR-223-3p	GTCGTATCCAGTGCAGGGTCCGAGGTATTCGCACTGGATACGACGGGG
U6-F	GGAACGATACAGAGAAGATTAGC
U6-R	TGGAACGCTTCACGAATTTGCG
TGFBR3-F	CCCTAACCGTGATGGGCATT
TGFBR3-R	TTCCTGCTGTCTCCCCTGT
Y-globin-F	GCAGCTTGTCACAGTGCAGTT
Y-globin-R	TGGCAAGAAGGTGCTGACTTC
β-actin-F	CAGGCACCAGGGCGTGAT
β-actin-R	TAGCAACGTACATGGCTGGG
miR-223-3p mimics-F	UGUCAGUUUGUCAAAUACCCCA
miR-223-3p mimics-R	GGGUAUUUGACAAACUGACAUU
miR223-3p mimics-NC-F	UUGUACUACACAAAAGUACUG
miR223-3p mimics-NC-R	GUACUUUUGUGUAGUACAAUU
miR223-3p inhibitor	(mU)(mG)(mG)(mG)(mG)(mU)(mA)(mU)(mU)(mU)(mG)
	(mA)(mC)(mA)(mA)(mA)(mC)(mU)(mG)(mA)(mC)(mA)
miR223-3pinhibitor-NC	(mC)(mA)(mG)(mU)(mA)(mC)(mU)(mU)(mU)(mU)(mG)
	(mU)(mG)(mU)(mA)(mG)(mU)(mA)(mC)(mA)(mA)

F, forward; R, reverse.

### Bioinformatics analysis and target gene prediction

The Arraystar miRNA expression profile microarray raw data was screened using *P*-value <0.05 and FC >1.5 as criteria. The mRNAs targeting miRNA were predicted using StarBase online tool (http://starbase.sysu.edu.cn/index.php). The target genes were screened based on comprehensive analysis of related functions and pathways of protein and verified by qRT-PCR.

### Cell culture

K562 cell lines (MeilunBio, Dalian, China) were cultured in RPMI 1640medium (Gibco, USA) containing 10% fetal bovine serum (FBS) and 1% penicillin–streptomycin. The cell lines were maintained at 37 °C in humidified incubator with 5% CO_2_. Cells in logarithmic growth with 95% viability were selected for subsequent experiments [14].

### Bioinformatics prediction and dual-luciferase reporter assay

The Starbase database predicted that miR-223-3p had a binding site with TGFBR3. The 293 T cells were transferred to 96-well plates overnight to reach a density of 50–70%. The sequence of miR-223-3p binding to TGFBR3 (seed sequence) was cloned and inserted into luciferase reporter plasmid (WT) by double-enzymatic cleavage, and an Ingenuclease reporter plasmid mutated by the seed sequence (MUT) was constructed at the same time. MiR-223-3p mimic or negative control (NC) was co-transfected into the cells together with the wild-type or mutant plasmid, respectively. Luciferase activity from the collected cell lysate was quantified using a Dual-Luciferase Reporter Assay Kit (Hanbio Biotechnology, ShangHai, China) and microplate reader. The interaction of miR-223-3p with TGFBR3 was analyzed based on the ratio between renilla luciferase activity and firefly luciferase activity.

### Cell induction and transfection

K562 cells were differentiated into the erythroid lineage by adding 50 μmmol/L hemin (Hemin chloride, Solarbio, Beijing, China), and the morphology and color of the cells were observed using microscope. The K562 cells were transfected with miR-223-3p mimic, miR-223-3p inhibitor, mimic negative control (mimic NC), and inhibitor NC using Entranster^TM^-R4000 (Engreen Biosystem Co, Ltd.) as the experimental groups, while K562 cells without transfection were set as the control group. Fluid exchange treatment was performed 24 h post transfection, and subsequent experiments were performed 48 h post transfection. The specific siRNA sequences are shown in Table 2.

### Measurement of mean corpuscular hemoglobin (MCH) and hemoglobin (HGB)

K562 cells were harvested following induction, and cell counts were performed for each treated group to eliminate the influence of differences in cell numbers on the experimental results. After washed with PBS, the cells were added with 100 μ1 of cell lysate and fully lysed on ice for 30 min, and then centrifuged at 12000 rpm for 10 min. The absorbance value was determined at 414 nm, and the MCH content of K562 cells was calculated based on its hemoglobin concentration of 130 μg/μl at 414 nm equal to 1.0. The cells were treated with the same method as above, and then 40 μl of benzidine working solution and 40 μl of 1% H_2_O_2_ were added to the cells and avoided light for 30 min. After added 10% glacial acetic acid, HGB content could be obtained by detecting the absorbance value at 490 nm with ELISA instrument.

### Cell proliferative assay

Transfected cells were seeded into 96-well plate (100 ul per well) at a concentration of 1x10^5^/ml. After incubated for 48 h, the cells were added with 10 ul of CCK-8 solution and continued to incubate for 2 h. Absorbance values of each well were measured at 450–490 nm (OD values), and the cells were then treated every 12 h.

### Flow cytometric

Analysis K562 cells were planted in 6-well plates at a density of 5x10^5^/ml. The transfected cells were rinsed with pre-cooled PBS, collected after digested by adding trypsin, resuspended with PBS, and then measured after adding PI and Annexin V-FITC antibodies. A Beckman flow cytometer (CytoflexS) was employed to measure apoptosis rates.

### Western blot analysis

RIPA Lysis Buffer (Servicebio) was added to K562 cells to obtain total protein samples. The protein concentration of the samples was determined using the BCA Protein Assay Kit (Solarbio). The protein concentrations were adjusted to be consistent among different groups. Then, the proteins were separated by SDS-PAGE and transferred to PVDF membrane. The membranes were incubated with primary antibodies including TGFBR3 (1:1500, Cell Signaling), Smsd2/3, P-Smad2, caspase-3, bcl-2 (1:2000, Abcam, Cambridge, MA, U.S.A.), and β-actin (1:2000, Servicebio) at 4 °C for 12 h. After thorough removal of the primary antibodies, the membrane was incubated with goat anti-rabbit secondary antibody (1:10000, Servicebio) at room temperature for 2 h. Protein expression was detected by chemiluminescence, and Image J software was used to process gray values and calculate relative protein expression.

### Statistical analysis

GraphPad Prism (version 8.0.1, La Jolla, CA, USA) was adopted for statistical analysis. The data were presented as mean ± SD or mean ± SEM, Differences between groups were compared using t-tests, while differences among multiple groups were compared using analysis of variance. *p* < 0.05 indicated statistical significance. All experiments were conducted independently at least three times.

## Results

### MiRNA and mRNA profiles determined by microarrays

**Figure 1.**
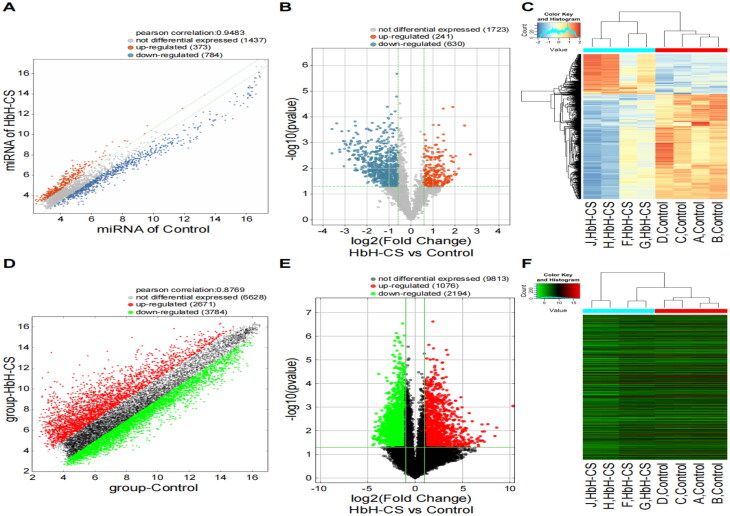
Comparison of the expression profiles of miRNAs(A-C) and mRNAs (D-F) between HbH-CS patients and healthy controls. (A) Scatter plot showing the distribution of miRNA expression. (B) Volcano plot showing the differential expression of miRNAs. (C) The clustering heatmap showed differentially expressed miRNAs between patients with HbH-CS patients and healthy controls. (D) Scatter plot showing the distribution of mRNA expression. (E) Volcano plot showing the differential expression of mRNAs. (F) The clustering heatmap showed differentially expressed mRNAs between patients with HbH-CS patients and healthy controls.

To identify miRNAs and mRNAs associated with HbH-CS disease, we studied miRNAs and mRNAs in 4 pairs of healthy subjects and HbH-CS disease by microarray. ArrayStar Human miRNA Array and ArrayStar Human mRNA Array were used to analyze the expression of miRNAs and mRNAs. The FC of their expression was determined and the results were that 871 miRNAs showed differential expression with a FC ≥1.5 and 3270 mRNAs showed differential expression with a FC ≥1.5. There were significant differences in expression between the HbH-CS disease and healthy cohorts (*p* < 0.05). Of these, 241 miRNAs were up-regulated, 630 miRNAs were down-regulated, 1076 mRNAs were up-regulated, and 2194 mRNAs were down-regulated, respectively (Figure 1A–1F).

### Identification of target miRNA and mRNA

**Figure 2.**
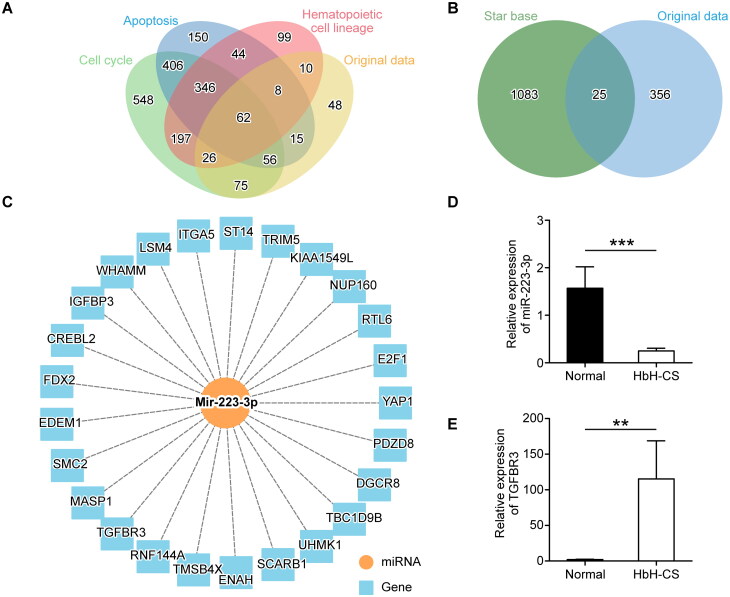
Bioinformatics analysis. (A) Venn diagram showed stratified operations of miRNAs from the original data and online database. (B) Intersection plot of mRNAs from our previous ArrayStar human mRNA array and miR-223-3p target gene predicted by online database. (C) Prediction plot of miR-223-3p target gene. Yellow circled node, miR-223-3p; blue rectangle type node, mRNA. (D, E) The qRT-PCR was performed to detect the relative expression levels of miR-223-3p (D) and TGFBR3 (E) in the samples from healthy normal subjects and HbH-CS patients. Normal group, *n* = 17; HbH-CS group, *n* = 17, mean ± SEM, **p < 0.01, ***p < 0.001.

We categorized the miRNA based upon online databases to identify miRNA with the most pleiotropic effects. A total of 972 miRNAs related to hematopoietic cell lineages, 1087 miRNAs related to apoptosis, and 1716 miRNAs related to the cell cycle were retrieved from the database. Using the Venn diagram, the top 300 differentially expressed miRNAs from the original data of miRNA expression profiles with the most pleiotropic effects were intersected, resulting in the further selection of 62 overlapping genes (Figure 2A). Next, the differentially expressed miRNAs were validated in 17 pairs HbH-CS patients and healthy clinical samples using qRT-PCR. The results showed that the expression of miR-223-3p was significantly down-regulated in HbH-CS patients (*p* < 0.05), which was consistent with the down-regulation trend observed in the original microarray data (Figure 2D). Using the StarBase online tool (http://starbase.sysu.edu.cn/index.php), target genes of miR-223-3p were predicted, which were intersected with the original microarray data from ArrayStar Human mRNA Array (Figure 2B and C). Finally, the target gene TGFBR3, a receptor for transforming growth factor β (TGF-β) superfamily, was identified based on the functional relevance of proteins and its pathways. Meanwhile, we found that TGFBR3 was differentially expressed between healthy subjects and HbH-CS patients in clinical sample validation (*p* < 0.05) (Figure 2E). Therefore, miR-223-3p/TGFBR3 was identified as the target of this study.

### MiR-223-3p interacted with TGFBR3-3’ UTR

**Figure 3.**
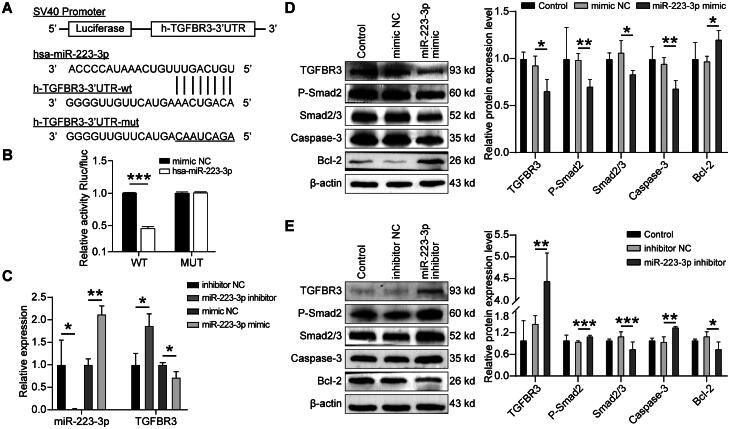
Overexpressing of miR-223-3p suppressed expression of TGFBR3. (A) Sites of miR-223-3p binding to the TGFBR3-3’UTR. (B) Dual luciferase reporter gene assay was performed to detect constructs cotransfected with control or miR-223-3p mimics. (C) The qRT-PCR was performed to detect miR-223-3p and TGFBR3 expression levels in K562 cells transfected with miR-223-3p inhibitor or miR-223-3p mimics. (D, E) Western blot analysis of TGFBR3, Smad2/3, P-Smad2, caspase-3, and bcl-2 in K562 cells transfected with miR-223-3p mimics, miR-223-3p inhibitor, or negative control, results are shown as mean ± SD from three independent experiments. *p < 0.05, **p < 0.01.

MiR-223-3p was predicted to bind to the 3′ UTR of TGFBR3 using miRNA target prediction tools, including Starbase [15], Targetscan, miRDB, miRanda, miRWalk, and DIANA, and bioinformatics analysis also showed that TGFBR3-3′ UTR has a putative matching seed sequence with miR-223-3p (Figure 3A). A dual-luciferase reporter assay was carried out to verify whether miR-223-3P targets 3′ UTR of TGFBR3. The results showed that wild-type TGFBR3 cotransfected with miR-223-3p mimic significantly decreased the luciferase activity, whereas the inhibitory effect of mutant TGFBR3 cotransfected with miR-223-3p mimic was not obvious (Figure 3B). Therefore, the results suggested that miR-223-3p could specifically bind to the seed region in the 3′ UTR of TGFBR3.

### MiR-223-3p regulated TGFBR3 expression in K562 cells

To further confirm the effect of miR-223-3p on TGFBR3, we used the miR-223-3p-specific inhibitor and miR-223-3p mimics to knock down and overexpress miR-223-3p expression in K562 cells, respectively. The results of qRT-PCR showed that knockdown of miR-223-3p could significantly up-regulated the mRNA levels of TGFBR3, whereas overexpression of miR-223-3p could lead to a significant decrease in mRNA expression of TGFBR3 (*p* < 0.05) (Figure 3C). Similar changes were observed in protein levels. Western blot showed that expression of TGFBR3 protein increased with repression of miR-223-3p and TGFBR3 protein expression decreased with overexpression of miR-223-3p (Figure 3D and E).

### MiR-223-3p regulated the TGFBR3/smad signaling pathway in K562 cells

Our results had confirmed that TGFBR3 was up-regulated in reticulocytes from HbH-CS patients (Figure 2E). Therefore, we hypothesized that increased TGFBR3 expression might lead to alteration in the TGF-β signaling pathway, which plays important role in hematopoietic process. To address this issue, we analyzed the activation of the typical TGF-β signaling pathway in K562 cells transfected with miR-223-3p-specific inhibitor and miR-223-3p mimics. The results showed that knockdown of miR-223-3p significantly increased the expression levels of Smad2/3 and P-Smad2 that is involved in erythropoiesis, whereas overexpression of miR-223-3p reduced the expression levels of Smad2/3 and P-Smad2 (Figure 3D and E). The change of Smad2/3 and P-Smad2 expression levels was consistent with that of TGFBR3. Thus, our results suggested that miR-223-3p may regulate erythropoiesis by mediating the TGFBR3/Smad signaling pathway.

### MiR-223-3p regulated cell proliferation and apoptosis

**Figure 4.**
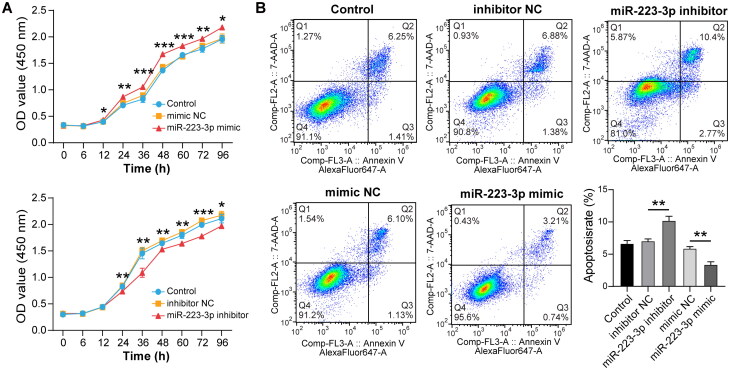
Detection of K562 cell proliferation and apoptosis (A) cell proliferation was evaluated by CCK8 assay in K562 cells transfected with miR-223-3p mimics, miR-223-3p inhibitor, or negative control. (B)Apoptosis rate was detected by flow cytometry, and cells were stained with annexin V-FITC and PI, Q2 refers to late-apoptotic cells. *p < 0.05, **p < 0.01. Results are shown as mean ± SD from three independent experiments.

To further investigate the biological function of miR-223-3p, we further performed cell proliferation and apoptosis experiments by transfecting with miR-223-3P mimics and miR-223-3p inhibitor into K562 cells. According to the results of CCK8 experiments, overexpression of miR-223-3p significantly enhanced the proliferation ability of the cells from the 12th hour, while repression of miR-223-3p inhibited the proliferation ability of cells from the 24th hour (Figure 4A). Cell counts were conducted, and the cell count of each group was 5,000 cells per well at 0 h. After 96 h, the cell counts in each group showed different degrees of change: the miR-223-3p mimic group had 33,000 cells per well, the mimic NC group had 21,400 cells per well, the miR-223-3P inhibitor group had 15,500 cells per well, and the inhibitor NC group had 23,000 cells per well. Apoptosis flow cytometry experiments also demonstrated that down-regulation of miR-223-3p impaired cell proliferation ability, whereas up-regulation of miR-223-3p inhibited cell apoptosis rate (Figure 4B). Our results indicated that up-regulation of miR-223-3p could promote cellular proliferation and reduce cellular apoptosis, which may play an important role in mediating erythropoiesis. To further explore the effect of miR-223-3p on apoptosis and its potential mechanism, we detected the expression levels of apoptosis-related proteins caspase-3 and bcl-2. The results showed that overexpression of miR-223-3p led to a decrease in caspase-3 expression level and an increase in bcl-2 expression level. Conversely, expression level of caspase-3 increased with repression of miR-223-3p, while the expression level of bcl-2 was significantly decreased (Figure 3D and E). Our study revealed the effects of miR-223-3p on cell proliferation and apoptosis.

### MiR-223-3p affected the levels of HGB and MCH

**Figure 5.**
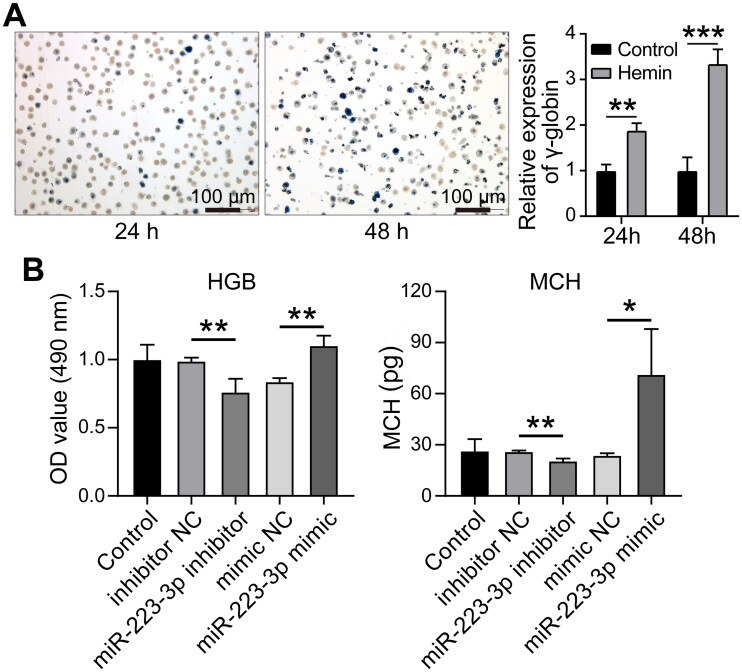
Relative expression levels of hemoglobin (HGB) and mean corpuscular hemoglobin (MCH) in K562 cells. (A) The expression of γ-globin in K562 cells induced toward the erythroid lineage was detected by qPCR at 24 and 48 h in both the blank group and the hemin group. (B) The levels of HGB and MCH were detected in K562 cells transfected with miR-223-3p mimics, miR-223-3p inhibitor, or negative control, *p < 0.05, **p < 0.01, ***p < 0.001. Each experiment was repeated three times. All data were shown in form of mean ± SD and error bars represent standard deviation.

In order to further explore the effect of miR-223-3p on HGB and MCH, we induced K562 cells into erythroid lineage with hemin, and the conversion rate could reach 37% and 70% at 24h and 48h, respectively (Figure 5A). Compared with the control group, the contents of HGB and MCH in induced K562 cells were significantly increased with overexpression of miR-223-3p. Opposite situations were observed when miR-223-3p was down-regulated in K562 cells (Figure 5B). These results indicated that miR-223-3p might influence on hemoglobin synthesis by targeting TGFBR3.

## Discussion

HbH-CS disease is a distinct thalassemia syndrome with a more severe clinical performance than other HbH disease form [16]. The HbCS is an α-globin chain variant caused by a mutation in the termination codon of the α2-globin gene with a very low yield of extended α-globin chain, thus leading to ineffective erythropoiesis [7,8]. The physiological process of erythropoiesis is complex and sophisticated, which is regulated by the cellular microenvironment, growth factors, transcriptional regulators, non-coding RNAs, etc [16,17]. MiRNAs are regulators of physiological processes by binding to the corresponding sequence region of target mRNA to regulate post-transcription. Evidences showed that miRNAs play important roles in erythroid homeostasis [18,19]. However, the role of miRNAs in HbH-CS disease remains unclear. To the best of our knowledge, this is the first study to identify differentially expressed miRNAs in patients with HbH-CS disease using microarray hybridization. The study identified a total of 871 differentially expressed miRNAs with 241 miRNAs up-regulation and 630 miRNAs down-regulation.

Further bioinformatics analysis and qRT-PCR verification in larger clinical samples were performed, and the results showed that miR-223-3p expression levels were significantly decreased in samples from HbH-CS disease compared to healthy controls. Previous studies demonstrated that miR-223 expressed in the bone marrow and participated in the bone marrow hematopoiesis [20]. MiR-223-3p, an important family member of miR-223, is involved in multiple important physiopathological processes. Previously, studies have showed that low expression of miR-223-3p was involved in the proliferation and invasion of various tumor cells, and it can influence on the occurrence of diseases by targeting genes and activating multiple signaling pathways [21,22]. Thus, we speculated that miR-223-3p might mediate target gene regulation and affect erythropoiesis in HbH-CS disease.

Dual-luciferase reporter gene assay confirmed that miR-223-3p directly interacted with TGFBR3, which was verified to be elevated in the patients with HbH-CS disease by qRT-PCR analysis in a larger sample. Studies have demonstrated that mutations in TGFBR3 can lead to abnormal generation process and function of erythrocytes [23] and significantly decrease the level of hemoglobin [24]. In addition, TGFBR3 and TGF-β signaling play key roles in differentiation of erythroid progenitor cells and erythropoiesis [25]. Erythropoiesis is a finely regulated process, and the proliferation, differentiation, and maturation of erythroid progenitor cells are controlled by various cytokines [26]. TGF-β superfamily signal transduction is involved in multiple biological processes such as cell quiescence, apoptosis, proliferation, differentiation, and migration [27,28], and has inhibitory effect on bone marrow activity, which can inhibit erythroid differentiation by inducing apoptosis of erythroblasts and stagnation of the cell cycle and play an important role in regulating the hemopoietic process [23,29,30]. TGFBR3, as a co-receptor of TGF-β, mediates TGF-β signaling. In the TGF-β signaling pathway, TGFBR3 regulates the stabilization of the receptor complexes of TGFBR1 and TGFBR2 with TGF-β by presenting TGF-β to TGFBR2, thereby mediating Smad pathway signaling [31,32]. Studies demonstrated that TGF-β superfamily members regulated erythropoiesis *via* the Smad pathway [33–35], and phospho-Smad2/3 combined with Smad4 inhibited progenitor cellular proliferation or promoted erythroid differentiation in combination with TIF1-γ [36]. Smad2/3 and P-Smad2 are involved in erythropoiesis. In this study, we further explored the effect of miR-223-3p on the TGF- β signaling pathway in K562 cells. K562 is a cell line of embryonic/fetal globin gene expression pattern, which can be induced to differentiate into erythroid lineage. The *in vitro* experiments showed that overexpression of miR-223-3p down-regulated the expression levels of TGFBR3, Smad2/3, and P-Smad2, significantly promoted cellular proliferation, and reduced cellular apoptosis; whereas repression of miR-223-3p up-regulated TGFBR3, Smad2/3, and P-Smad2 expression, inhibited the proliferation ability of cells, and promoted cell apoptosis. These results were consistent with a previous report that suppression of miR-223-3p inhibited proliferation and induced apoptosis. Collectively, these findings suggest that miR-223-3p may regulate erythropoiesis by mediating TGFBR3/Smad signaling pathway in HbH-CS disease.

According to the literature, HbH-CS patients are classified as having a more severe clinical phenotype of α-thalassemia, and a reduction in hemoglobin levels is one of its distinguishing features [8,37,38]. Single nucleotide polymorphisms in miRNA genes or their target genes may play a role in causing aberrant expression of hemoglobin [19]. The decrease of hemoglobin in HbH-CS disease may be caused by increase in erythrocyte apoptosis. A previous report demonstrated that the abnormal expression of TGFBR3 gene had significantly lower levels of hemoglobin [24]. Consistent with previous finding, our study identified that repression of miR-223-3p resulted in a significant decrease of hemoglobin level in cells with up-regulation of TGFBR3. Overexpression of miR-223-3p increased hemoglobin level by suppressing expression of TGFBR3. We cannot rule out the possibility that the observed increase in hemoglobin levels is associated with the overexpression of miR-223-3p in cells and its protective effects.

However, this study has limitations. Unfortunately, due to restricted experimental conditions, we were unable to cultivate primary cells as a better model for HbH-CS disease. However, previous research suggested that K562 cells, as widely available erythroid precursors, could withstand extensive transfection experiments and exhibit excellent performance *in vitro*, providing researchers with a reliable and stable experimental cell model [39]. Many studies on thalassemia have utilized K562 cells as a surrogate model, and the studies indicate comparable results between K562 cells and primary cells [39–42].

## Conclusions

In conclusion, we identified firstly that existence of numerous differentially expression miRNAs in patient with HbH-CS disease compared to healthy subjects. Our findings demonstrated that miR-223-3p reciprocally regulated TGFBR3/Smad signaling pathway, and overexpression of miR-223-3p promoted cellular proliferation, inhibited apoptosis, and increased hemoglobin levels. Overall, the present study preliminarily elucidated the molecular mechanism of miR-223-3p in HbH-CS disease and provided new therapeutic targets for treatment of patients with HbH-CS disease.

## Data Availability

All raw data of this study are available from the corresponding author by request.
